# Decontamination and reuse of surgical masks and N95 filtering facepiece respirators during the COVID-19 pandemic: A systematic review

**DOI:** 10.1017/ice.2020.379

**Published:** 2020-07-30

**Authors:** Kachorn Seresirikachorn, Vorakamol Phoophiboon, Thitiporn Chobarporn, Kasenee Tiankanon, Songklot Aeumjaturapat, Supinda Chusakul, Kornkiat Snidvongs

**Affiliations:** 1 Department of Otolaryngology, Faculty of Medicine, Chulalongkorn University, Bangkok, Thailand; 2 Endoscopic Nasal and Sinus Surgery Excellence Center, King Chulalongkorn Memorial Hospital, Bangkok, Thailand; 3 Division of Pulmonology and Critical Care Medicine, Department of Medicine, Faculty of Medicine, Chulalongkorn University, Bangkok, Thailand; 4 Excellence Center for Critical Care Medicine, King Chulalongkorn Memorial Hospital, Thai Red Cross Society, Bangkok, Thailand; 5 Department of Surgery, Faculty of Medicine, Chulalongkorn University, Bangkok, Thailand; 6 Division of Gastroenterology, Chulalongkorn University and King Chulalongkorn Memorial Hospital, Thai Red Cross Society, Bangkok, Thailand

## Abstract

**Objectives:**

Surgical masks and N95 filtering facepiece respirators (FFRs) prevent the spread of severe acute respiratory syndrome coronavirus-2 (SARS-CoV-2) infection and protect medical personnel. Increased demands for surgical masks and N95 FFRs during the coronavirus disease 2019 (COVID-19) pandemic has resulted in the shortage crisis. However, there is no standard protocol for safe reuse of the N95 FFRs. In this systematic review, we aimed to evaluate the effectiveness of existing decontamination methods of surgical masks and N95 FFRs and provide evidence-based recommendations for selecting an appropriate decontamination method.

**Methods:**

We performed systematic searches of Ovid MEDLINE and Ovid EMBASE electronic databases. The last search was performed April 11, 2020. Any trials studying surgical masks and/or N95 FFRs decontamination were included. Outcomes were disinfections of virus and bacteria, restoration of the filtration efficiency, and maintenance of the physical structure of the mask.

**Results:**

Overall, 15 studies and 14 decontamination methods were identified. A low level of evidence supported 4 decontamination methods: ultraviolet (UV) germicidal irradiation (9 studies), moist heat (5 studies), microwave-generated steam (4 studies), and hydrogen peroxide vapor (4 studies). Therefore, we recommended these 4 methods, and we recommended against use were given for the other 10 methods.

**Conclusions:**

A low level of evidence supported the use of UV germicidal irradiation, moist heat, microwave-generated steam, and hydrogen peroxide vapor for decontamination and reuse of N95 FFRs. These decontamination methods were effective for viral and bacterial disinfection as well as restoration of the filtration efficiency, and the physical structure of the FFRs.

Coronavirus disease 2019 (COVID-19) is a highly contagious disease, caused by severe acute respiratory syndrome coronavirus 2 (SARS-CoV-2).^1^ The SARS-CoV-2 virus is transmitted through respiratory droplets^2^ and close contact with infected individuals.^3^ Aerosolized particles generated by medical procedures such as transsphenoidal endoscopic pituitary surgery could be another transmission route.^4^ Healthcare providers taking care of COVID-19 patients without appropriate personal protective equipment (PPE) are at high risk for infection. A shortage of surgical masks has resulted from an abrupt rise in global demand.^5^ While surgical masks filter infectious particles spreading via droplets, filtering facepiece respirators (FFRs) filter >95% of airborne particles. These masks are designed for single use.^6^ Reuse of these disposable masks has been implemented during the COVID-19 pandemic,^7^ although the appropriate method of decontamination remains unclear. Concerns include sterility, filtration efficiency, and structural integrity.^8,9^ In this systematic review, we assessed the evidence of various decontamination methods of surgical masks and FFRs, including N95 and P100.

## Methods

### Eligibility criteria

This systematic review followed the Preferred Reporting Items for Systematic Reviews and Meta-Analyses (PRISMA)^10^. We applied the following inclusion criteria: trials studying the performance of decontamination and reuse of surgical masks and/or FFRs, any study designs, any device, any methods, and any models of FFRs. We also applied the following exclusion criteria: studies published in a language other than English, nonexperimental studies, and studies without original data. The outcome measures were disinfection of bacteria and virus, post-decontamination filtration efficiency, and physical structure degradation.

### Information sources and search strategy

Electronic systematic searches were conducted. The last search was performed on April 11, 2020. Literature searches were performed using Ovid MEDLINE and Ovid EMBASE. We also scanned references of the included studies to identify any missing published or unpublished trials. We used the following search strategy: (“exp Respiratory Protective Devices/ or Filtering Facepiece Respirators.mp.” or “exp Respiratory Protective Devices/ or N95.mp.” or “face mask.mp.” or “exp Masks/ or surgical mask.mp.” or “medical masks.mp.”) and (“decontamination.mp. or exp Decontamination/” or “exp Recycling/ or reuse.mp.” or “reusability.mp.”).

### Study selection and data collection

Two review authors (V.P. and T.C.) independently performed trial selection by title and abstract screening based on predetermined eligibility criteria. The full-text articles of the selected studies were reviewed for the final study selection. Two authors (K.Se. and K.T.) extracted data from the included studies. Disagreements were resolved by the fifth author (K.Sn.).

## Results

**Fig. 1.**
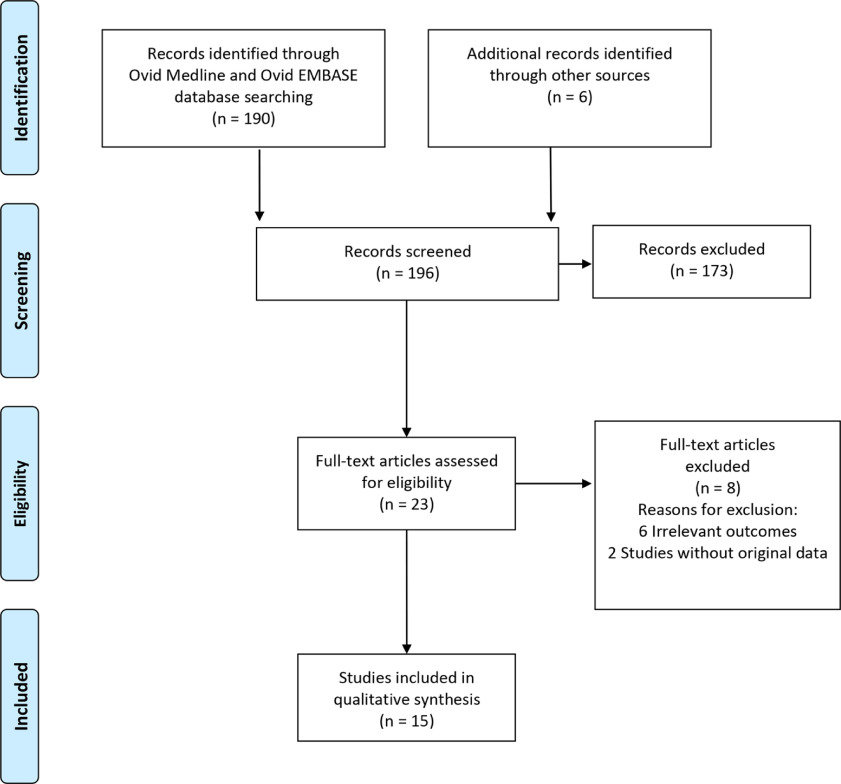
Flow diagram of study selection for the systematic review.

We identified 196 studies: 190 studies from electronic searches, and 6 studies from manual searches. During the title and abstract screening, 173 studies were irrelevant and excluded. After full-text screening, 8 studies were excluded. Finally, 15 studies were included in the qualitative synthesis (Fig. 1).^8,11–24^

### Included studies

None of the 15 included studies assessed surgical masks. All studies assessed FFRs, including N95 and P100. All included studies were nonhuman subject research. Of the 15 studies, 4 studies (27%) assessed disinfection of bacteria,^12,17,21,22^ 7 studies (47%) assessed disinfection of virus,^8,11,13–15,18,24^ 9 studies (60%) assessed postdecontamination filtration efficiency,^11,12,15,16,19–22,24^ and 12 studies (80%) assessed physical structure degradation.^8,11–14,16,19–24^

We identified 14 decontamination methods. Data regarding ultraviolet germicidal irradiation (UVGI) (9 studies)^8,11–18^ are described in Supplementary Table 1 (online). Data regarding moist heat (5 studies)^8,12–15^ are described in Supplementary Table 2 (online). Data regarding microwave-generated steam (MGS) (4 studies)^8,13–15^ and hydrogen peroxide vapor (HPV) (4 studies)^12,21–23^ are described in Supplementary Table 3 (online). Data regarding microwave steam bags (1 study),^24^ bleach (5 studies),^11,12,17,19,20^ steam treatment, (3studies),^17,19,20^ dry heat (3 studies),^17,19,20^ ethanol or isopropyl alcohol (3 studies)^17,19,20^, ethylene oxide (EtO) (3 studies),^11,12,19^ hydrogen peroxide gas plasma (HPGP) (2 studies),^11,19^ liquid hydrogen peroxide (LHP) (2 studies),^12,19^ microwave irradiation (1 study),^19^ and soap and water (1 study)^19^ are described in Supplementary Table 4 (online).

### Ultraviolet germicidal irradiation

Overall, 9 studies assessed the performance of UVGI decontamination method.^8,11–18^ All studies evaluated ultraviolet light-C (UV-C) with a primary wavelength of 254 nm. However, a great variety of equipment and delivery techniques were employed: a laminar-flow cabinet with UV-C light,^11,13–15^ a UV-C lamp,^8,12,17^ and a chamber with a UV-C bulb.^16,18^ Diversity across studies included the intensity of UV-C (W/cm^2^), the dose of UV-C (J/cm^2^), the distance between the source of UV-C and the FFRs, the exposure surface of the FFRs, the total exposure time, and the number of cycles. The exposure time per cycle varied from 1 minute,^16–18^ 15 minutes,^8,13,15^ 20 minutes,^17^ 30 minutes^11,14^ to 45 minutes.^12^ The number of cycles varied from 1^8,11,14–18^ to 3 cycles.^12,13^

UVGI was effective for influenza virus inactivation, including H5N1 (2 studies)^11,15^ and H1N1 (3 studies),^8,13,18^ and *Bacillus subtilis* spore inactivation.^17^ Post-decontamination filtration efficiency was unchanged in 4 studies.^11,12,15,16^ The physical structure was unchanged in 3 studies,^11–13^ but physical strength partially lost with high doses of UV-C at 120 J/cm^2^ and 950 J/cm^2^ and the head strap strength lost at 590 J/cm^2^.^16^ The optimal UV-C dose should be <2 J/cm^2^. Laminar flow cabinet was suggested for 3M 1860, 3M 1870, Kimberly Clark PFR 95-270.^11,13–15^ UV-C chamber was suggested for 3M 1860, 3M 9210, Gerson 1730, Kimberly Clark 46727.^16,18^ UV-C lamp was effective, but the FFRs model was unspecified.^8,12,17^

### Moist heat

In 5 studies, the performance of moist heat decontamination method was assessed,^8,12–15^ and 2 types of equipment were used. In 3 studies, N95 FFRs were decontaminated with a laboratory incubator for a 30-minute incubation at 60°C. The FFRs were air dried after each incubation: overnight after the first incubation and for 30 minutes after the second and the third incubations.^12–14^ In 2 studies, a 6-L sealable container filled with 1 L tap water was warmed in a 65°C oven for a minimum of 3 hours. Then the FFRs were placed on a rack to isolate the FFRs from the liquid, and the containers were sealed and returned to the oven.^8,15^ The exposure time per cycle ranged from 15 minutes^13^ to 20 minutes^15^ to 30 minutes.^8,12,14^ The number of cycles ranged from 1^8,14,15^ to 3.^12,13^

Moist heat was effective for the H1N1^8^ and H5N1^15^ influenza virus inactivation when using a prewarmed sealable container. Viral inactivation was not achieved by a laboratory incubator. Bacterial disinfection was not assessed. The postdecontamination filtration efficiency was >97.5%.^12,15^ The physical structure was reported unchanged by 1 study^8^ but degradation was reported in some models by 3 studies.^12–14^ A 6-L prewarmed sealable container filled with 1 L tap water was suggested for models 3M 1860 and 3M 1870.^8,15^

### Microwave-generated steam

In 4 studies, the performance of MGS decontamination was assessed.^8,13–15^ FFRs were placed outer-side down on top of 2 side-by-side pipette tip boxes with 50 mL room-temperature tap water, in a 1,100W, 1,250W (2,450 MHz) microwave oven with a revolving glass carousel. The exposure time was 2 minutes at the maximum power setting. Then the FFRs were dried overnight on a laboratory benchtop. In 3 studies, the FFRs were decontaminated with 1 cycle,^8,14,15^ and in 1 study FFRs were decontaminated with 3 cycles.^13^ MGS inactivated >4-log reduction of the viable virus.^8,13–15^ Bacterial disinfection was not assessed. The post-decontamination filtration efficiency was unchanged.^15^ In 3 studies a slight separation of the inner-foam nose cushion was observed in some samples.^8,13,14^ Although a minor physical structure degradation was reported, the FFRs had a 90%–100% fit-test passing rate.^13^ A 1,250W (2450 MHz) microwave oven with a revolving glass carousel was suggested for 3M 1860.^8,15^

### Hydrogen peroxide vapor

In 4 studies, the performance of HPV decontamination was assessed for 3, 5, 30, and 50 cycles (225, 405, and 480, and 1,440 minutes per cycle).^12,21–23^ An HPV generator utilizing 30% or 35% hydrogen peroxide solution was placed in a room. The FFRs were placed on stainless-steel wire racks. The HPV run consisted of the following 5 stages: conditioning, pre-gassing, gassing, gassing dwell, and aeration. The processing room attained the 480+ parts per million (ppm) level of HPV with gassing times of 25 and 40 minutes and gassing dwell times of 15, 20, and 25 minutes (ie, the sterilization process). During the aeration stage, fresh air was introduced into the room to increase the rate of catalytic conversion of hydrogen peroxide into water and oxygen.^12,21–23^ In addition, 4 hours of aeration eliminated the toxicity of hydrogen peroxide.^22^ Viral disinfection was not assessed. HPV was effective for *Geobacillus stearothermophilus* spore inactivation.^12,21,22^ Post-decontamination filtration efficiency^21,22^ and the physical structure were unchanged.^12,22,23^ Placement of the FFRs on stainless-steel wire racks in the room with an HPV generator was suggested for 3M 1860.^21,22^

### Microwave steam bags

One study assessed the performance of microwave steam bag decontamination.^24^ The FFRs were placed inside separate bags filled with 60 mL tap water. The bags were sealed, placed in a microwave oven, and irradiated on high power for 90 seconds. Bacteriophage MS2, a surrogate for a pathogenic virus, was thereby inactivated, but bacterial inactivation was not assessed. The postdecontamination filtration efficiency and the physical structure were unchanged.^24^

### Bleach

The performance of bleach decontamination was assessed in 5 studies.^11,12,17,19,20^ The FFRs were submerged in a 0.6% aqueous solution of sodium hypochlorite for 1–3 cycles. The exposure time ranged from 10 to 30 minutes.^11,12,17,19,20^ After treatment, they were hung on a laboratory pegboard and allowed to air dry overnight. Virus inactivation was not assessed. Bleach was effective for *Bacillus subtillis* spore inactivation.^17^ The postdecontamination filtration efficiency was unchanged in 3 studies,^11,12,19^ but it decreased in 1 study.^20^ The physical structure of N95 FFRs was degraded after the 30 minutes of decontamination.^11,12,19^

### Steam

The performance of steam decontamination was assessed in 3 studies.^17,19,20^ Characteristics of these studies assessing steam are described in Supplementary Table 5 (online). The FFRs were sealed in an autoclave bag and treated in an autoclave at 121°C. The FFRs were air dried for 72 hours. Virus inactivation was not assessed. Steam was effective for *Bacillus subtillis* spore inactivation.^17^ The filtration efficiency decreased in 2 studies,^19,20^ and the outer layer of the N95 FFRs was deformed, shrunken, and stiff.^19,20^

### Dry heat

The performance of dry heat decontamination was assessed in 3 studies^17,19,20^ using 2 types of equipment. One study used a hot-air oven; respirators were placed in a metal pan on racks of a laboratory oven and were turned over midway through the exposure period (60 minutes) for 1 cycle at 80°C and 160°C.^19^ Two studies used an electric rice cooker at 149–164°C for 3 minutes for 1 cycle,^17,20^ but virus inactivation was not assessed. Dry heat with an electric cooker was effective on the disinfection of *Bacillus subtilis* spores.^17^ The postdecontamination filtration efficiency was unchanged, but the FFRs were melted at 160°C after 22 minutes of decontamination.^19^

### Ethanol or isopropyl alcohol

The performance of ethanol or isopropyl alcohol decontamination was assessed in 3 studies.^17,19,20^ Ethanol with various concentrations and volumes was added to the center of the surface of the N95 FFRs. The FFRs were then dried in a petri dish placed in a biosafety cabinet for 10 minutes, followed by another 10 minutes of submersion in 100% isopropanol solution. Virus inactivation was not assessed. Ethanol was effective in the disinfection of *Bacillus subtilis* spores.^17^ The postdecontamination filtration efficiency decreased,^19,20^ and the physical structure was unchanged.^19,20^

### Other methods

Other decontamination methods assessed neither viral nor bacterial inactivation. Post-decontamination filtration efficiency remained unchanged for EtO,^11,12,19^ HPGP,^11,19^ LHP,^12,19^ microwave irradiation decontamination.^19^ In contrast, soap and water decreased filtration efficiency.^19^ Physical degradation was shown in the ethylene oxide, HPGP, LHP, microwave irradiation methods^11,12,19^ but the physical structure was unchanged for soap and water.^19^

## Discussion

None of the existing published articles had data on the SARS-CoV-2 disinfection. However, both the influenza virus and the SARS-CoV-2 are in the same group of lipid bilayer enveloped viruses.^25–27^ Therefore, the data on the decontamination of the influenza virus could be applied to the COVID-19 setting. The studies assessed *Bacillus subtilis* ^17^ and *Geobacillus stearothermophilus* ^12,21,22^ disinfection. Spores of these bacteria are more challenging to disinfect than viruses; thus, the data can be applied to the COVID-19 pandemic.^28–30^ Bacteriophage MS2, a surrogate for a pathogenic virus, was also assessed. Fisher et al^24^ reported that steam bags were 99.9% effective in inactivating MS2 on the FFRs. However, they commented that more research was required before the data could be applied.

**Fig. 2.**
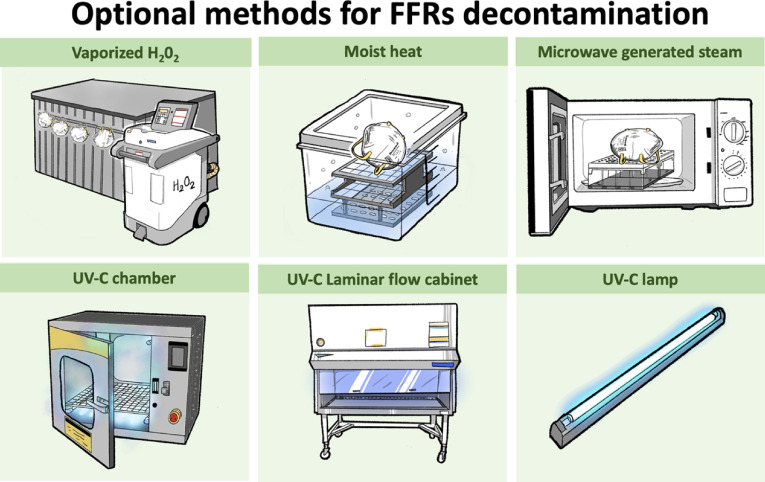
Optional methods for FFRs decontamination.

We recommend 4 decontamination methods as options in response to a preponderance of benefit over harm shown by nonhuman subject research: UVGI,^8,11–18^ moist heat,^8,12–15^ MGS,^8,13–15^ and HPV,^12,21–23^ UVGI,^8,11–18^ moist heat,^8,12–15^ MGS^8,13–15^ and HPV.^12,21–23^ These methods were effective in disinfecting virus and bacteria and in maintaining the filtration efficiency and the physical structure of the FFRs (Fig. 2). We do not recommend other decontamination methods for 3 reasons. (1) Several methods did not assess the virus and bacteria disinfection: microwave steam bag,^24^ EtO,^11,12,19^ HPGP,^11,19^ LHP,^12,19^ microwave irradiation,^19^ and soap and water.^19^ (2) Several methods decreased the filtration efficiency: soap and water,^19^ ethanol and isopropyl alcohol,^19,20^ and microwave irradiation.^19^ And (3) several methods destroyed the physical structure of the masks: bleach,^11,12,19^ HPGP,^11,19^ and microwave irradiation.^11,19^ A summary of the performance of the 14 decontamination methods is displayed in Supplementary Table 5 (online).

FFRs = filtering facepiece respirators; UV-C = ultraviolet light-C; H2O2 = hydrogen peroxide.

The UV-C light decontaminates viruses by damaging the DNA and RNA of the virus. A study by Darnell et al^31^ showed that the UV-C light source (254 nm), which emitted 4.016 W/cm^2^ at a distance of 3 cm for 15 minutes, could inactivate severe acute respiratory syndrome coronavirus 1 (SARS-CoV-1) virus. For clinical applicability, the UV-C concentration, a distance between the UV-C light and the masks, and exposure time must be considered. The findings from this review showed that the UV-C light exposure at 1.6–2.2 W/cm^2^ for 1–3 cycles (15–30 minutes per cycle) could inactivate the H1N1 and H5N1 influenza viruses, maintain filtration efficiency, and restore the physical structure of the FFRs.^8,11–18^ Heat inactivates viruses by modifying the protein structures of the virus that affects the attachment and replication within a host cell. Heat at 65°C inactivates most SARS-CoV-1 after 4 minutes.^31^ One cycle of moist heat exposure using a sealable container for 20 minutes inactivates the H5N1 virus^15^ and the H1N1 after 30 minutes.^8^ Physical structure degradation may occur when the temperature is >60–70°C or when >1 cycle is used.^12–14^ Mid-to-high relative humidity increases viral inactivation, although 100% humidity is not effective.^32,33^ The SARS-CoV-1 infectivity is reduced by 60–75°C heat exposure in various liquid media.^31^ McDevitt et al^34^ showed that H1N1 inactivation in a dried solution on stainless steel when either temperature or relative humidity was increased.^34^ Therefore, MGS was recommended as an option. Although a minor physical structure degradation was reported, the FFRs passed a fit test.^8,13,14^ The combination of hydrogen peroxide gas and the generation of hydroxyl and hydroperoxyl free radicals inactivates spores of the *Geobacillus stearothermophilus* bacteria.^12,21,22^ Compared to other decontamination methods, HPV can increase the number of cycles up to 20 cycles and still maintain filter efficiency and physical structure.^21^ The Battelle Decontamination System, an HPV system for decontaminating N95 masks, received emergency use authorization from the FDA on March 28, 2020.^35^

Our recommendations align with the Centers for Disease Control and Prevention (CDC) recommendations about the emergency reuse of UVGI, moist heat, and HPV. Furthermore, the CDC suggests that healthcare workers should have at least 5 pieces of N95 FFRs and that the used FFRS should be kept in a breathable paper bag and labeled at the end of each shift. The FFRS should be reused with a minimum of 5 days after the last use.^35^ This recommendation is based on the study by van Doremalen et al^36^ showing that SARS-CoV-2 can survive for up to 72 hours on plastic, stainless steel, and cardboard surfaces. In contrast, Chin et al^37^ found that the SARS-CoV-2 could be detected on the outer layer of a surgical mask after 7 days. We believe that no good evidence supports the safety of the reuse of medical masks after keeping the used masks for 72–98 hours.^35^

Although surgical masks are not indicated to protect general people from the transmission of respiratory pathogens, masks are overused by the public and surgical masks are scarce. The reuse of masks was not recommended in normal situations. The masks were not manufactured for multiple uses; they were not intended for extended wear and should not be worn for several hours at a time. Medical personnel should follow the recommended reuse techniques summarized in Supplementary Table 5 (online). Otherwise, the masks could lose their filtering efficiency, which could lead to a failure of protection against infection. A decontaminated mask is not a fresh mask. After each decontamination, a seal check should always be conducted before wearing the mask. The mask must fit with the face with no leaking point for letting the air out. The straps should be intact and must not be loose. If a mask loses its structure, it should be discarded immediately. Touching the inside surface of the mask should be avoided. After touching the mask, the hands must be washed with soap and water for at least 20 seconds or sanitized using a hand rub with at least 60% alcohol.^6^ The reused mask should be worn by the same person.

The limitation of this study was the quality of the included studies. No clinical study has proven that the studied methods are clinically effective. We detected heterogeneity among the included studies, with considerable variation in decontamination equipment and techniques. The volume of masks was not addressed. Currently, no data are available on methods for disinfection of SARS-CoV-2. Instead, studies investigating influenza virus and bacteria spores were included here. High-quality studies investigating SARS-CoV-2 decontamination from used surgical masks are required for a higher level of evidence in future research.

In conclusion, decontamination of surgical masks and N95 FFRs is necessary to prepare them for reuse in the shortage crisis during the COVID-19 pandemic. The selection of decontamination methods should be considered based on the data in which the effectiveness of virus and bacterial disinfection, the filtration efficiency, and the intact physical structure of the masks and FFRs after the decontamination process. Based on the influenza virus and bacterial inactivation, the UVGI, moist heat, MGS, and HPV methods were recommended as options. When these decontamination methods are used in practice, the techniques described in the literature should be strictly followed.

## Supplementary material

For supplementary material accompanying this paper visit http://dx.doi.org/10.1017/ice.2020.379. click here to view supplementary material
