# Supplementary material for: Decontamination and reuse of surgical masks and N95 filtering facepiece respirators during the COVID-19 pandemic: A systematic review
Source: Infect Control Hosp Epidemiol. 2020 Jul 30:1–6. doi: 10.1017/ice.2020.379 (PMC7438629; doi:10.1017/ice.2020.379)
Supplement: Supplementary file 1 [file S0899823X20003797sup001.docx]

| **Author**  **(Year)** | **Mask (Model)** | **Equipment** | **Methods** | **No. of cycles** | **UV-C intensity**  **(mW/cm^2^)** | **Times**  **(mins)** | **Disinfection** | | **Filtration efficiency^a^** | **Physical structure^b^** |
| --- | --- | --- | --- | --- | --- | --- | --- | --- | --- | --- |
|  |  |  |  |  |  |  | **Bacteria** | **Virus** |  |  |
| Viscusi et al.^11^  (2009) | N95 (N95-A, N95-B, N95-C), Surgical N95 (SN95-D, SN95-E, and SN95-F), P100 (P100-G, P100-H, P100-I) | Laminar flow cabinet | -FFRs were placed in a laminar flow cabinet with a 40 Watts UV-C light  -Total of a 30-mins exposure | 1 | 0.18-0.2 | 15/ each side | N/A | Inactivated (H5N1 Influenza) | No change | No change |
| Bergman et al.^12^  (2010) | N95 (N95-A, N95-B, N95-C),  Surgical N95 (SN95-D, SN95-E, SN95-F) | UV-C lamp | -FFRs were placed 25 cm below 40 Watts UV-C lamp.  -Total of a 45-mins exposure | 3 | 1.8 | 45 | N/A | N/A | No change | No change |
| Heimbuch et al.^8^  (2011) | N95,  Surgical N95 | UV-C lamp | -FFRs were placed 25 cm below of a 120 cm, 80 Watts UV-C lamp.  -Total of 15-mins exposure at the temperature of 22±2 ^o^C and humidity of 75±5% | 1 | 1.6-2.0 | 15 | N/A | Inactivated (H1N1 Influenza) | N/A | N/A |
| Viscusi et al.^14^  (2011) | N95 (3M 8000, 3M 8210, Moldex 2200, 3M 1860,3M 1870, Kimberly Clark PFR95–270) | Laminar flow cabinet | -FFRs were placed in laminar flow cabinet with a 40 Watts UV-C bulb.  -Total of a 30-mins exposure | 1 | 1.8 | 15/ each side | N/A | N/A | N/A | Moldex 2200 strap broke |
| Bergman et al.^13^  (2012) | N95 (3M 1860, 3M 1870, Kimberly Clark PFR 95-270 (46767)) | Laminar flow cabinet | -FFRs were placed in a laminar flow cabinet with a 40 Watts UV-C bulb.  -Total of a 15-mins exposure | 3 | 1.6-2.0 | 15 | N/A | Inactivated (H1N1 Influenza) | N/A | No change |
| Lore et al.^15^  (2012) | N95 (3M 1860, 3M 1870) | Laminar flow cabinet | -FFRs were placed in a laminar flow cabinet (126 Lx15.2Wx10.8H cm^3^) with dual-bulb, 15 Watts UV-C lamps.  -The lamp was 25 cm above FFRs.  -Total of a 15-mins exposure | 1 | 1.6-2.2 | 15 | N/A | Inactivated (H5N1 Influenza) | No change | N/A |
| Lindsley et al.^16^  (2015) | N95 (3M 1860, 3M 9210, Gerson 1730, Kimberly Clark 46727) | UV-C custom chamber | -FFRs were placed in a UV-C custom chamber (91x31x64 cm^3^) with 6.2 cm distance.  - Dual-bulb, 15 Watts UV-C lamps  -Total of 1-min exposure | 1 | 2000-39,333 | 1 | N/A | N/A | No change | -Physical strength partially lost at 120 J/cm^2^ and totally lost at 950 J/cm^2^.  - Head strap strength lost at 590 J/cm^2^. |
| T.-H. Lin et al.^17^  (2018) | N95 | UV-C lamp | FFRs were placed 10 cm below 6 Watts UV-C lamp. | 1 | 18.9 | 1,2,5,10,20 | Inactivated (*Bacillus subtilis* spore) | N/A | N/A | N/A |
| Mills et al.^18^  (2018) | N95  (3M 1860, 3M 1870, 3M Vflex1805  Alpha Protech 695, Gerson 1730, Kimberly-Clark PFR, Moldex 1512, Moldex 1712, Moldex EZ-22, Precept 65-3395, Prestige Ameritech RP88020, Sperian HC-NB095, Sperian HC-NB295F, U.S. Safety AD2N95A, U.S. Safety AD4N95) | UV-C custom device | -FFRs were placed in a UV-C polished aluminum device.  -Total of 1-min exposure at the temperature of 21±2 ^o^C and humidity of 48±6% | 1 | 16.67 | 1 | N/A | Inactivated (H1N1 Influenza) | N/A | N/A |

**Table 1 Characteristics and results of UVGI decontamination method’s studies.**

No.= number; UV-C= ultraviolet light-C; mW/cm^2^= milliwatts per square centimeter; mins= minutes; FFRs= filtering facepiece respirators; N/A= not applicable; cm= centimeter; ^o^C= degree Celsius; L= length; W= Width; H= Height; cm^3^= cubic centimeter; J/cm^2^= Joules per square centimeter

^a^ Filtration efficiency after decontamination

^b^ Physical structure of FFRS after decontamination

| **Study**  **(Year)** | **Mask (Model)** | **Equipment** | **Methods** | **No. of Cycles** | **Temperature**  **(°C)** | **Times**  **(mins)** | **Disinfection** | | **Filtration efficiency^a^** | **Physical structure^b^** |
| --- | --- | --- | --- | --- | --- | --- | --- | --- | --- | --- |
|  |  |  |  |  |  |  | **Bacteria** | **Virus** |  |  |
| Bergman et al.^12^  (2010) | N95 (N95-A, N95-B, N95-C),  Surgical N95 (SN95-D, SN95-E, SN95-F) | Caron model 6010 laboratory incubator (Marietta, Ohio) | 30-mins incubation at 60°C, 80% relative humidity in a Caron model 6010 laboratory incubator (Marietta, OH). | 3 | 60 | 30 | N/A | N/A | >97.5% | All SN95-E samples experienced partial separation of the inner foam nose cushion from the FFRs. |
| Viscusi et al.^14^  (2011) | N95 (3M 8000,  3M 8210, Moldex 2200, 3M 1860, 3M 1870, Kimberly  Clark PFR95–270) | Caron Model 6010 laboratory  incubator (Marietta, Ohio) | 30-mins incubation at 60°C, 80% relative humidity in a Caron model 6010 laboratory incubator (Marietta, OH). | 1 | 60 | 30 | N/A | N/A | N/A | -3M 1870 experienced a slight separation of the inner foam nose cushion  -Moldex 2200 strap broke and detached from the staple. |
| Heimbuch et al.^8^  (2011) | N95,  surgical N95 | 6 Litre sealable container (17Hx19Wx19L cm^3^) | -The container was filled with 1 Litre of tap water. A plastic support rack was placed in the water to isolate the FFRs from the liquid.  -The container was warmed in an oven to 65 ± 5°C for 3 hours, and it was removed from the oven.  -An H1N1-contaminated FFRs were placed on the rack. The containers were sealed and returned to the oven for 30 mins. | 1 | 65 ± 5 | 30 | N/A | > 4-log reduction of viable H1N1 Influenza | N/A | No change |
| Bergman et al.^13^  (2012) | N95 (3M 1860, 3M 1870, Kimberly Clark PFR 95-270 (46767)) | Caron model  6010 laboratory incubator (Marietta, Ohio) | 15-mins incubation at 60 °C (upper temp. limit), 80 % relative humidity in a Caron model 6010 laboratory incubator (Marietta, OH). | 3 | 60 | 15 | N/A | N/A | N/A | - The fit test was 90-100%.  - 3M 1870 experienced a slight separation of the inner foam nose cushion. |
| Lore et al.^15^  (2012) | N95 (3M 1860 and 3M 1870) | 6 Litre sealable container (17Hx19Wx19L cm^3^) | -The container was filled with 1 Litre of tap water. A plastic support rack was placed in the water to isolate the FFRs from the liquid.  -The container was warmed in an oven to 65 ± 5°C for 3 hours, and it was removed from the oven.  -An H1N1-contaminated FFRs were placed on the rack.  -The containers were sealed and returned to the oven for 20 mins. | 1 | 65 ± 5 | 20 | N/A | > 4-log reduction of viable H5N1 Influenza | 3M 1860s: 98.96%,  3M 1870: 99.01% | N/A |

**Table 2 Characteristics and results of moist heat decontamination method’s studies.**

No.= number; ^o^C= degree Celsius; mins= minutes; N/A= not applicable; FFRs= filtering facepiece respirators; cm= centimeter; L= length; W= Width; H= Height; cm^3^= cubic centimeter

^a^ Filtration efficiency after decontamination

^b^ Physical structure of FFRS after decontamination

| **Study**  **(Year)** | **Mask (Model)** | **Equipment** | **Methods** | **No. of Cycles** | **Temperature**  **(°C)** | **Times**  **(mins)** | **Disinfection** | | **Filtration efficiency^a^** | **Physical structure^b^** |
| --- | --- | --- | --- | --- | --- | --- | --- | --- | --- | --- |
|  |  |  |  |  |  |  | **Bacteria** | **Virus** |  |  |
| **Decontamination method for N95 respirator using MGS** | | | | | | | | | | |
| Viscusi et al.^14^  (2011) | N95 (3M 8000, 3M 8210, Moldex 2200, 3M 1860, 3M 1870, Kimberly  Clark PFR95–270) | 2450 MHz, Sharp Model R-305KS (Sharp Electronics, Mahwah, N.J.) microwave oven with revolving glass carousel, 1100 Watts (manufacturer rated) | -750 W/ft^3^ experimentally measured; 2 mins total exposure at a power setting of 10 (maximum power). FFRs placed outer-side down on top of two side-by-side pipette tip boxes, centered, (each box 11.7x8x5cm^3^) with 50 ml room temperature tap water (∼20°C)  -Following treatment, FFRs dried overnight on a laboratory benchtop. | 1 | N/A | 2 | N/A | Reduced by > 4 logs | N/A | One 3M 1870 and one Moldex 2000 samples had slight separation of the foam nose cushion and broken strap. |
| Heimbuch et al.^8^  (2011) | N95,  Surgical N95 | 1250 Watts microwave oven | -Two plastic reservoirs (12x38x4.5 cm^3^) with perforated tops (192 holes of 6 mm diameter, spaced uniformly over the entire surface) was filled with 50 ml of tap water at 228^◦^C-258^°^C.  -The reservoirs were placed together, and the contaminated FFRs were set atop the center of the assembly, with the exterior of the FFRs resting on the surface of the reservoir.  -The reservoir assembly and FFRs were loaded into the center of a 1250-Watts microwave oven and irradiated at full power for 2 mins. | 1 | N/A | 2 | N/A | Reduced by > 4 logs | N/A | Some samples had a slight separation of the foam nose cushion. |
| Bergman et al.^13^  (2012) | N95 (3M 1860, 3M 1870, Kimberly Clark PFR 95-270 (46767)) | 2450 MHz, Sharp Model R-305KS (Sharp Electronics, Mahwah, N.J.) microwave oven with revolving glass carousel, 1100 Watts (manufacturer rated) | -750 W/ft^3^ experimentally measured; 2 mins total exposure at a power setting of 10 (maximum power).  -FFRs placed outer-side down on top of two side-by-side pipette tip boxes, centered, (each box 11.7x 8x5 cm^3^) with 50 ml room temperature tap water (∼20°C). | 3 | N/A | 2 | N/A | Reduced by > 4 logs | N/A | -The fit test was 90-100%.  -3M 1870 experienced a slight separation of the inner foam nose cushion.  -One head strap was  melting in a Kimberly Clark PFR95-270 sample during the third treatment. |
| Lore et al.^15^  (2012) | N95 (3M 1860, 3M 1870) | A 1250-Watts (2450 MHz) commercially available microwave oven (Panasonic Corp., Secaucus, NJ, USA) with a rotating  glass plate | -FFRs were placed above a plastic box filled with 50 ml of room temperature tap water. The top of the box was perforated with 96 holes (7 mm diameter) evenly distributed over the entire surface to allow MGS to vent through the respirator.  -The virus-contaminated respirator was placed with the convex surface pointed toward  the steam source, and the FFRs were then irradiated for 2 mins at full power. | 1 | N/A | 2 | N/A | > 4-log reduction of viable H5N1 Influenza | No Change | N/A |
| **Decontamination method for N95 respirator using HPV** | | | | | | | | | | |
| Bergman et al.^12^  (2010) | N95 (N95-A, N95-B, N95-C),  Surgical N95 (SN95-D, SN95-E, SN95-F) | Room Bio-Decontamination Service (RBDSTM, BIOQUELL UK Ltd, Andover, U.K.) which contains 4 portable modules: the Clarus-R HPV generator, the Clarus R20 aeration unit, an instrumentation module, and a control computer | -FFRs were hung on a string stretching across the length of the room. Starting15-mins dwell with 125-mins total cycle time in Clarus-R HPV generator (utilizing 30% hydrogen peroxide). -Following HPV exposure, the Clarus R20 aeration unit was run overnight inside the room to convert the HPV into oxygen and water vapor catalytically. | 3 | N/A | 1,440 | *Geobacillus stearothermophilus* spores | N/A | Slightly decreased but remained above 95%. | No change |
| Final report for the Bioquell HPV decontamination^21^ (2016) | N95 (3M 1860) | Bioquell Clarus^TM^ C system HPV generator | One HPV cycle included 10 mins conditioning phase, 20 mins gassing phase at 2 g/min, 150 mins dwell phase at 0.5 g/min, and a 300 mins aeration phase | 50 | N/A | 480 | *Geobacillus stearothermophilus* spores | N/A | Not affected over the 50 cycles | The elastic material in the straps fragmented when stretched after 30 cycles |
| Schwartz et al.^22^ (2020) | N95 (3M 1860) | Bioquell Clarus^TM^ C system HPV generator with 35% hydrogen peroxide solution | - Place the FFRs on stainless steel wire racks in the room with Bioquell Clarus^TM^ C system.  - Production of HPV at a concentration of 480+ ppm took first 25 mins for “gassing” time the sterilization process began and lasted for 20 mins.  - The last step was aeration for 4 hours for conversion hydrogen peroxide to water and oxygen that could eliminate the toxicity of hydrogen peroxide. | 30 | N/A | 285 | *Geobacillus stearothermophilus* spores | N/A | No change | No change |
| Kenney et al.^23^ (2020) | N95 (3M 1870, 3M St. Paul, MN) | BQ-50 system (Bioquell, Horsham, PA) | - Hanging by their elastic on racks in a room and sterilized with BQ-50 using a 10 mins conditioning phase, 30-40 mins gassing phase, 25 mins dwell phase, and a 150 mins aeration phase (varies with a number of respirators and room size).  - 50% steam sterilization at 135 ^o^C for 5 mins | 5 | 135 | 215-225 | N/A | N/A | N/A | No change |

**Table 3 Characteristics and results of MGS and HPV decontamination method’s studies.**

No.= number; ^o^C= degree Celsius; mins= minutes; MGS= microwave generated steam; MHz= Megahertz; W/ft^3^= Watts per cubic foot; cm^3^= cubic centimeter; ml= milliliter; N/A= not applicable; FFRs= filtering facepiece respirators; mm= millimeter; HPV, hydrogen peroxide vapor; g/min= gram per minute; ppm= parts per million

^a^ Filtration efficiency after decontamination

^b^ Physical structure of FFRS after decontamination

| **Study**  **(Year)** | **Mask (Model)** | **Equipment** | **Methods** | **No. of Cycles** | **Temperature**  **(°C)** | **Times**  **(mins)** | **Disinfection** | | **Filtration efficiency^a^** | **Physical structure^b^** |
| --- | --- | --- | --- | --- | --- | --- | --- | --- | --- | --- |
|  |  |  |  |  |  |  | **Bacteria** | **Virus** |  |  |
| **Decontamination method for N95 respirator using microwave steam bag** | | | | | | | | | | |
| Fisher et al.^24^  (2011) | N95 (3M 1860, 3M 1870, 3M8210,  Kimberly-Clark  PFR95, Moldex 2200, Cardinal Health N95) | Medela Quick Clean^TM^/  MICRO-STEAMTM BAGS (Medela, McHenry, IL) /MunchkinH Steam Guard^TM^ Bags | The FFRs were placed inside separate bags filled with 60 ml of tap water. The bags were sealed, placed in a microwave oven, and irradiated on high power for 90 seconds. | 3 | N/A | N/A | N/A | 99.9% effective for inactivating MS2 | Slightly decreased but remained above 95%. | No change |
| **Decontamination method for N95 respirator using bleach** | | | | | | | | | | |
| Viscusi et al.^19^  (2007) | N95, P100 | Fisher 5.25% Sodium Hypochlorite (NaOCl) with 0.20% Sodium Hydroxide (NaOH) | 4 or 8 FFRs were placed (submerged) into a dishpan or 4 litres beaker containing 3-5 litres of treatment solution (0.525% and 5.25%). | 1 | N/A | 30 | N/A | N/A | The average penetration was not significantly increased. | The aluminum nosebands were tarnished (0.525% and 5.25%), stiffening of filter media, and elastic straps (5.25%). |
| Viscusi et al.^11^  (2009) | N95 (N95-A, N95-B, N95-C), Surgical N95 (SN95-D, SN95-E, and SN95-F), P100 (P100-G, P100-H, P100-I) | The solution of sodium hypochlorite (original concentration was 6% available as Cl_2_) | 30-mins submersion in 0.6% (one-part bleach to nine parts of deionized water) solution of sodium hypochlorite | 1 | N/A | 30 | N/A | N/A | Reached expected levels of filter aerosol penetration  and filter airflow resistance | - Metallic nosebands were slightly tarnished and visibly not shiny.  - SN95-E inner nose comfort cushion was discolored.  All FFRs were dry to the touch and had a smell of bleach. |
| Bergman et al.^12^  (2010) | N95 (N95-A, N95-B, N95-C),  Surgical N95  (SN95-D, SN95-E, SN95-F) | The solution of sodium hypochlorite (original concentration was 6% available as Cl_2_) | 30-mins submersion in 0.6% (one-part bleach to nine parts of deionized water) solution of sodium hypochlorite | 3 | N/A | 30 | N/A | N/A | >97.5% (except  SN95-D ;>95%) | - Metallic nosebands were slightly tarnished and visibly not shiny.  -Staples were oxidized to varying degrees.  - All FFRs were dry to the touch and had a smell of bleach. |
| Tzu-Hsien Lin et al.^20^  (2017) | N95,  Gauze,  Spunlace-nonwoven | 0.5% sodium hypochlorite solution (original concentration was 0.5% available as Cl_2_). | 10 mins submersion in 0.5% sodium hypochlorite solution | 1 | N/A | 10 | N/A | N/A | Decreased | N/A |
| T.-H. Lin et al.^17^  (2018) | N95 | 5.4% (weight/weight) as Cl_2_: original; 2.7%: one part bleach to one part of deionized water; 0.54%: one part bleach to nine parts of deionized water | 0.4 ml volume of bleach was added to the center of the surface of the N95 using a pipette.  - The FFRs were dried in a petri dish in a biosafety cabinet for 10 mins. | 1 | N/A | 10 | Inactivated  100%  (*Bacillus subtilis* spores) | N/A | N/A | N/A |
| **Decontamination method for N95 respirator using steam treatment** | | | | | | | | | | |
| Viscusi et al.^19^  (2007) | N95,  P100 | Market Forge Automatic Sterilmatic Steam Pressure Sterilizer (Everett, MA) | -All samples were sealed in a standard poly/paper autoclave bag and treated in a Market Forge.  -Automatic Sterilmatic Steam Pressure Sterilizer (Everett, MA) for 15 and 30 mins. | 1 | 121 | 15,30 | N/A | N/A | Significantly decreased for N95 at 15 and 30 mins, and significantly decreased for P100 at 15 mins. | N95 were deformed,  shrunken, stiff, and mottled.  There were no changes for P100. |
| Tzu-Hsien Lin et al.^20^  (2017) | N95,  Gauze,  Spunlace-nonwoven | Autoclave | Set the temperature at 121°C with 1.06 kg/cm^2^ for 15 mins. | 1 | 121 | 15 | N/A | N/A | Decreased | The outer layer of N95 was deformed, shrunken, and stiff. |
| T.-H. Lin et al.^17^  (2018) | N95 | Autoclave | FFRs were heated for 15 mins at 121°C and 103 kilopascals. | 1 | 121 | 15 | Inactivated almost  100% (*Bacillus subtilis* spore) | N/A | N/A | N/A |
| **Decontamination method for N95 respirator using dry heat** | | | | | | | | | | |
| Viscusi et al.^19^  (2007) | N95,  P100 | Hot air oven  (Fisher Isotemp 500  Series (Fisher Scientific, Pittsburgh, PA)) | -FFRs were placed in a metal pan on racks of a laboratory oven at 80°C and 160°C for 60 mins.  -Turned over midway through the exposure period. | 1 | 80,160 | 60 | N/A | N/A | No change | -There were no changes at 80°C after 60 mins.  - Melted at 160 °C after 22 mins. |
| Tzu-Hsien Lin et al.^20^  (2017) | N95,  Gauze,  Spunlace-nonwoven | Traditional electric rice cooker (Taiwan) | -Place the test of FFRs decontamination in a traditional electric rice cooker using dry heat for 3 mins (149-164˚C, without adding water). | 1 | 149-164 | 3 | N/A | N/A | Decreased | N/A |
| T.-H. Lin et al.^17^  (2018) | N95 | Traditional electric rice cooker (Taiwan) | -The FFRs were placed in an electric rice cooker for dry heating for 3 mins (149-164°C, without added water). | 1 | 149-164 | 3 | almost  100%  (Bacillus subtilis  spores) | N/A | N/A | N/A |
| **Decontamination method for N95 respirator using EtO** | | | | | | | | | | |
| Viscusi et al.^19^ (2007) | N95, P100 | Steri-Vac 4XL (3M, St. Paul, MN, USA) and Steri-Vac 5XL (3M, St. Paul, MN, USA) | -Steri-Vac 4XL sterilizer processed in the warm cycle of 55° C and 883 mg/L EtO gas and Steri-Vac 5XL sterilizer processed in the warm cycle of 55° C and 725 mg/L EtO.  -Four samples were placed in standard poly/paper pouches and treated with EtO.  -All samples were exposed to EtO for 1-hour, followed by a 4-hours of aeration. | 1 | 55 | 300 | N/A | N/A | Slightly changed | P100 straps were darkened slightly. EtO 3M 5XL was found to be slightly less degrading than EtO 3M 4XL. |
| Viscusi et al.^11^  (2009) | N95 (N95-A, N95-B, N95-C),  Surgical N95 (SN95-D, SN95-E, SN95-F),  P100 (P100-G, P100-H, P100-I) | Steri-Vac 5XL sterilizer (3M, St Paul, MN, USA) | - FFRs and a chemical indicator placed in a single warm cycle (55°C and 725 mg/L 100% EtO gas).  -EtO exposure was 1hour, followed by 4-hours of aeration. | 1 | 55 | 300 | N/A | N/A | Not change | No change |
| Bergman et al.^12^  (2010) | N95  (N95-A, N95-B, N95-C),  Surgical N95 (SN95-D, SN95-E, SN95-F) | Amsco Eagle 3017 100% EtO Sterilizer/Aerat (STERIS Corp., Mentor, OH, USA) | -FFRs were packaged in Steris Vis-U-All Low-Temperature Tyvek/polypropylene-polyethylene Heat Seal Sterilization pouches and placed in 100% EtO Sterilizer/Aerator on HI-TEMP setting (55°C) with 1-hour of EtO exposure (736.4 mg/L), followed by 12-hours aeration. | 3 | 55 | 60 | N/A | N/A | Slightly decreased but remained above 95%. | No change |
| **Decontamination method for N95 respirator using ethanol and isopropyl alcohol** | | | | | | | | | | |
| Viscusi et al.^19^  (2007) | N95, P100 | Henry Shein Isopropyl  alcohol 70% | 1 second and 1 min in 70% isopropyl alcohol | 1 | N/A | 0.02,1 | N/A | N/A | Decreased | Fading of strap ink was the only visible change observed |
| Tzu-Hsien Lin et al.^20^  (2017) | N95, Gauze,  and Spunlace | 70% ethanol solution  Isopropyl alcohol | 10 mins of submersion in 70% ethanol solution. | 1 | N/A | 10 | N/A | N/A | Increased penetration of both 75 nm and  300 nm particles through the masks | No change |
| T.-H. Lin et al.^17^  (2018) | N95 | Ethanol with various concentration | Ethanol with various concentrations and volumes was added to the center of the surface of the FFRs using a pipette; the FFRs were then dried in a petri dish that was placed in a biosafety cabinet (BSC) for 10 mins. | 1 | N/A | 10 | Relative survival of Bacillus subtilis spores  was 73 ± 5% initially and decayed to 22 ± 8% in 24 hours | N/A | N/A | N/A |
| **Decontamination method for N95 respirator using HPGP** | | | | | | | | | | |
| Viscusi et al.^19^  (2007) | N95, P100 | STERRADNX Standard cycle and STERRAD 100S (Advanced Sterilization Products, Irvine, CA, USA) | N/A | 1 | - | - | N/A | N/A | No change | Aluminum nosebands were slightly tarnished and visibly not as shiny. |
| Viscusi et al.^11^  (2009) | N95 (N95-A, N95-B, N95-C),  Surgical N95 (SN95-D, SN95-E, and SN95-F) P100 (P100-G, P100-H, P100-I) | STERRAD 100S HPGP Sterilizer (Advanced Sterilization Products, Irvine, CA, USA) | Single of 55-mins standard cycle. FFRs and a chemical indicator placed in an individual Mylar/Tyvekä self-seal pouch. | 1 | - | 55 | N/A | N/A | No change | Metallic nosebands were slightly tarnished and visibly not as shiny when compared with their as-received counterparts. |
| **Decontamination method for N95 respirator using LHP** | | | | | | | | | | |
| Viscusi et al.^19^  (2007) | N95, P100 | 3% and 6% hydrogen peroxide solution | Submersion in 3% and 6% hydrogen peroxide for 30 mins | 1 | N/A | 30 | N/A | N/A | No significantly changed for the N95.  There was significantly more variable for the P100. | No change |
| Bergman et al.^12^  (2010) | N95 (N95-A, N95-B, N95-C),  Surgical N95 (SN95-D, SN95-E, SN95-F) | 30% hydrogen peroxide; Cat No. H325-500, CAS Nos. 7722-84-1, 7732-18-5, 12058-66-1 (Fisher Scientific, Fair Lawn, NJ). | 30-mins submersion in 6% solution of hydrogen peroxide | 3 | N/A | N/A | N/A | N/A | Slightly decreased but remained above 95%. But no data in actual filtration efficiency in bioaerosols. | LHP treatment caused staples to oxidize to a varying degree. |
| **Decontamination method for N95 respirator using microwave irradiation** | | | | | | | | | | |
| Viscusi et al.^19^  (2007) | N95, P100 | 2,450-MHz microwave oven 750W/ft^3^, Sharp Model R-305KS (Sharp Electronics, Mawwah, NJ) | FFRs was irradiated for half the time, promptly turned over, and  irradiation was repeated for the remainder of the allotted time. | 1 | N/A | 2,4 | N/A | N/A | - There was no change at 2 mins.  -N95 filter penetration was significantly increased at 4 mins.  -P100 was melted at 4 mins. | N95 filter media was melted at the ends of the aluminum nosebands and formed visible holes at 4 mins,  P100 face seal was melted at 4 mins |
| **Decontamination method for N95 respirator using soap and water** | | | | | | | | | | |
| Viscusi et al.^19^  (2007) | N95, P100 | Ivory bar soap and water solution with concentration 1 g/L | FFRs was submersed in the soap and water solution with concentration 1 g/L for 2 and 20  mins. | 1 | N/A | 2,20 | N/A | N/A | Average penetration was markedly  increased for N95 respirators at both time intervals.  In P100, increased in penetration at 2 mins, while the 20-mins treatment significantly increased average penetration | No change |

**Table 4 Characteristics and results of other decontamination method’s studies.**

No.= number; ^o^C= degree Celsius; mins= minutes; FFRs= filtering facepiece respirators; ml= milliliter; N/A= not applicable; kg/cm^2^= kilogram per square centimeter; EtO= Ethylene oxide; mg/L= milligram per liter; HPGP, hydrogen peroxide gas plasma; LHP, liquid hydrogen peroxide; MHz= Megahertz; W/ft^3^= Watts per cubic foot; g/L= milligram per liter

^a^ Filtration efficiency after decontamination

^b^ Physical structure of FFRS after decontamination

| **Decontamination methods** | **No.** | **Disinfection** | | **Filtration efficiency^b^**  **(No.)** | **Physical structure (No.)** | **LOE** | **Recommendation** | **Recommended techniques** |
| --- | --- | --- | --- | --- | --- | --- | --- | --- |
|  |  | **Bacteria (No.)** | **Virus (No.)** |  |  |  |  |  |
| Ultraviolet germicidal  irradiation ^8, 11-18^ | 9 | Inactivate almost 100% of *Bacillus subtilis* spore^17^ (1) | Inactivate  -H5N1 Influenza^11, 15^ (2)  -H1N1 Influenza^8, 13, 18^ (3) | No change^11, 12, 15, 16^ (4) | -No change^11-13^ (3)  -Physical strength loss at 120-950 J/cm^2 16^ (1)  -The strap broke for 1 model^14^(1) | 4 | Option | (1) A laminar flow cabinet delivering a UV-C light with a UV-C dose of 0.32-1.98 J/cm^2^ for 30 mins (15 mins to each surface) was suggested for the following FFRs models: 3M 1860, 3M 1870, Kimberly Clark PFR 95-270. (1-3 cycles).  (2) UV-C chamber, made of polished aluminum measuring 40x16x13 inches^3,^ delivering a UV-C dose of 1 J/cm^2^ for 1 min was suggested for the following FFRs models: 3M 1860, 3M 9210, Gerson 1730, Kimberly Clark 46727(1 cycle).  (3) UV-C lamp delivering a UV-C light with a UV-C dose of 1.44-1.98 J/cm^2^ was also effective, but the FFRs model was unspecified (1-3 cycles). |
| Moist heat^8, 12-15^ | 5 | N/A | Reduced > 4-log reduction of viable  -H1N1 Influenza^8^ (1)  -H5N1 Influenza^15^ (1) | >97.5%^12, 15^ (2) | - Fit test pass 90-100% ^12^ (1)  - Some models of N95 FFRs experience a slight separation of the inner foam nose cushion from FFRS^12-14^ (3) | 4 | Option | A 6-Litre sealable warmed container filled with 1-Litre of tap water was suggested. The FFRs were placed and isolated from the liquid before the containers were sealed and returned to the oven. One cycle was suggested with the exposure time of 20-30 mins at 65 ± 5 °C. It was suggested for the models 3M 1860, and 3M 1870. |
| Microwave generated steam^8, 13-15^ | 4 | N/A | - Reduced > 4- logs Reduction^8, 13, 14^ (3)  - Reduced > 4- logs reduction H5N1 Influenza^15^ (1) | No Change^15^ (1) | - Fit test pass 90-100%^13^ (1)  - Some models of N95FFRs experience a slight separation of the inner foam nose cushion from FFRs^8, 13, 14^ (3) | 4 | Option | A 1250-Watts (2450 MHz) microwave oven with revolving glass carousel at a maximum power setting with the exposure time of 2 mins for one cycle was suggested for the model 3M 1860. |
| Hydrogen peroxide vapor^12, 21-23^ | 4 | Inactivate  - *Geobacillus stearothermophilus* spores^12, 21, 22^ (3) | N/A | No Change^21, 22^ (2) | No change^12, 22, 23^ (3) | 4 | Option | Hang the respirator on a stainless wire in the room with an HPV generator. Production of HPV from 35% hydrogen peroxide at a concentration of 480+ ppm took time for first 25 mins for “gassing” time then the sterilization process began and lasted for 20 mins. 4-hour aeration for conversion hydrogen peroxide to water and oxygen was the last step to eliminate the toxicity of hydrogen peroxide. Twenty to thirty cycles were suggested for the model 3M 1860. |
| Microwave steam bags^24^ | 1 | N/A | Inactivate 99.9%  MS2^24^ (1) | >95%^24^ (1) | No change^24^ (1) | 4 | Recommendation against |  |
| Bleach^11, 12, 17, 19, 20^ | 5 | Inactivate almost 100% of *Bacillus subtilis* spore^17^ (1) | N/A | - >95%^11, 12, 19^ (3)  - Decrease^20^ (1) | Degradation^11, 12, 19^ (3) | 4 | Recommendation against |  |
| Steam treatment^17, 19, 20^ | 3 | Inactivate almost 100% of *Bacillus subtilis* spore^17^ (1) | N/A | Decrease^19, 20^ (2) | N95 were deformed, shrunken and stiff^19, 20^ (2) | 4 | Recommendation against |  |
| Dry heat^17, 19, 20^ | 3 | Inactivate almost 100% of *Bacillus subtilis* spore^17^ (1) | N/A | - No change^19^ (1)  - Decrease^20^ (1) | N95 melted at 160°C^19^ (1) | 4 | Recommendation against |  |
| Ethanol or Isopropyl alcohol^17, 19, 20^ | 3 | Inactivate 80% of  *Bacillus subtilis* spore in 24 hours^17^ (1) | N/A | -Decrease^19, 20^ (2) | No change^19, 20^ (2) | 4 | Recommendation against |  |
| Ethylene oxide^11, 12, 19^ | 3 | N/A | N/A | >95%^11, 12, 19^ (3) | Degradation^19^ (1)  - No change^11, 12^ (2) | 4 | Recommendation against |  |
| Hydrogen peroxide gas plasma^11, 19^ | 2 | N/A | N/A | No change^11, 19^ (2) | Slightly change of metallic noseband^11, 19^ (2) | 4 | Recommendation against |  |
| Liquid hydrogen peroxide^12, 19^ | 2 | N/A | N/A | No change^12, 19^ (2) | - Slight change of staples^12^ (1)  - No change^19^ (1) | 4 | Recommendation against |  |
| Microwave irradiation^19^ | 1 | N/A | N/A | - N95 was melted^19^ (1) | N95 was melted^19^ (1) | 4 | Recommendation against |  |
| Soap and water^19^ | 1 | N/A | N/A | Decreased^19^ (1) | No change^19^ (1) | 4 | Recommendation against |  |

**Table 5 Summary of the performance of fourteen decontamination methods.**

No.= number of studies; LOE= level of evidence; J/cm^2^= Joules per square centimeter; UV-C= ultraviolet light-C; mins= minutes; ^o^C= degree Celsius; FFRs= filtering facepiece respirators; MHz= Megahertz; ppm= parts per million

^a^ Filtration efficiency after decontamination

^b^ Physical structure of FFRS after decontamination

**Figure legends**

**Figure 1 Flow diagram of study selection for the systematic review.**

**Figure 2 Optional methods for FFRs decontamination.**

FFRs= filtering facepiece respirators; UV-C= ultraviolet light-C; H_2_O_2_= hydrogen peroxide
